# Clinical Advances in Hepatobiliary Surgery: Diagnosis, Prognosis, Management and Surgery for Colorectal Liver Metastases

**DOI:** 10.3390/jcm15093315

**Published:** 2026-04-27

**Authors:** Alexandros E. Giakoustidis, Vasileios N. Papadopoulos, Dimitrios E. Giakoustidis, Matteo Donadon, Guido Torzilli

**Affiliations:** 1 First Department of Surgery, General Hospital Papageorgiou, Aristotle University of Thessaloniki, 56429 Thessaloniki, Greece; papadvas@auth.gr (V.N.P.); dgiakoustidis@gmail.com (D.E.G.); 2 Surgical Oncology Program, University Maggiore Hospital, University of Piemonte Orientale, 28100 Novara, Italy; matteo.donadon@uniupo.it; 3 Division of Hepatobiliary Surgery & General Surgery, Humanitas Research Hospital, 20089 Rozzano, Italy; guido.torzilli@hunimed.eu

Colorectal liver metastases remain a fascinating field in the area of hepatobiliary diseases and surgery, with diagnosis, prognosis, management, and surgical treatment in an area of focus for the latest advancements. For this reason, for this Special Issue, we have brought together experts in the field as an editorial team guided by Professor Guido Torzilli, a leader in liver surgery. In partnership with Professors Vasileios N. Papadopoulos, Dimitris E. Giakoustidis, and Matteo Donadon, we have prepared a Special Issue with articles covering aspects of interest in this field.

Approximately 15% of patients with colorectal cancer will already have liver metastases at diagnosis. Advances in chemotherapy regimens over the past few decades have led to increased rates of disease regression, sometimes resulting in downstaging patients from inoperable to operable. However, in a number of patients with colorectal liver metastases, the hepatic lesions can be missing on preoperative imaging following neoadjuvant chemotherapy; thus, there is ongoing global debate on the surgical management of the sites of these disappearing liver metastases [1,2]. Papakontstantinou et al. provided a detailed systematic review on the subject, analyzing a total of 326 patients with 1134 disappearing liver metastases [3]. The analysis concluded that, despite the radiological advancements in liver metastasis assessment, there is a big proportion of patients with vanishing lesions in preoperative imaging and a high percentage being recognized intraoperatively. Of these lesions, a high percentage—67.51%—had viable cancer cells in postoperative histopathology analysis. Furthermore, a percentage of resected disappearing liver metastases based on the previous site only and not recognized during surgery still had viable cancer cells in histopathology analysis. This partly explains the recurrence of the disease at follow-up and justifies the debate on how to manage these lesions.

Moving on to another subject of interest, perihilar lymph node dissection is still a controversial topic in patients with colorectal liver metastases. Hess et al. explored the role of perihilar lymph node dissection in patients with colorectal liver metastases [4]. The incidence of regional LN metastases around the liver in patients with colorectal hepatic metastases can reportedly reach 14% after histologic examination [5,6], with debatable guidelines for performing lymph node dissection. In conclusion, the authors demonstrate that preoperative imaging is not sensitive enough to detect affected lymph nodes accurately and that CK22 staining helps identify micro-metastases and isolated tumor cells in resected lymph nodes of patients with colorectal liver metastases. Also, they state that perihilar lymph node dissection is not associated with better recurrence-free or overall survival in these patients.

On the other hand, a key parameter for better prognosis remains a clear surgical margin. Paramythiotis et al. examined the impact of negative surgical margins ranging from 1 to 10 mm and those of >10 mm on patient survival [7,8,9,10]. Six studies met the inclusion criteria, with the results of this meta-analysis revealing the superiority of wider surgical margins (>10) on overall survival compared to smaller margins (1–10 mm). The authors highlighted the importance of margin optimization in surgical management strategies.

One cannot refer to advances in hepatobiliary surgery without addressing technological developments, including robotic-assisted liver surgery [11,12,13]. Schiavone et al. assessed 14 studies including 771 patients, 62% of which underwent wedge and 38% underwent anatomical resections (21.39% major and 16.61% minor) [14]. Although the authors conclude that robotic-assisted liver resection is considered a well-established technique, further prospective and comparative studies with standardized outcome reporting are still needed to define optimal patient selection and long-term effectiveness.

Liver metastasis management does not only concern surgery, and with this in mind, Paramythiotis investigated non-surgical therapeutic approaches [15]. Specifically, histotripsy, which was recently approved by the Food and Drug Administration (FDA), seems to be promising for overcoming a number of limitations [16,17,18]. Among its advantages are the lack of thermal injury, its non-invasive nature, and its immunopreserving and possibly immunostimulating ability, as well as the low number of complications following its application. Of course, continued clinical evaluation is essential to validate its long-term efficacy and integration into standard care.

Last but not least, the training of future liver surgeons with 3D printed liver molds is highlighted [19,20]. Elisei et al. thoroughly investigated gelatin-based phantom liver recipes, noting that they are an inexpensive, reproducible, and accessible alternative for training surgeons in image-guided and diagnostic procedures and can meet most training requirements [21].

“Ever to excel”, or, in other words, always strive for excellence, is a motto characterizing all specialties dealing with colorectal liver metastases, and with this in mind, we finalize this Special Issue and look forward to the next.
